# A Mussel-Inspired Hydrogel Loaded with Ginsenoside Rh1 for Perianal Ulcer Healing

**DOI:** 10.3390/biomimetics11100726

**Published:** 2026-10-11

**Authors:** Chaorui Jiang, Zezhou Xu, Zhenglei Yu

**Affiliations:** 1 Weihai Institute for Bionic, Jilin University, Weihai 264402, China; jiangzr21@mails.jlu.edu.cn (C.J.);; 2 Key Laboratory of Bionic Engineering, Ministry of Education, Jilin University, 5988 Renmin Street, Changchun 130025, China

**Keywords:** mussel-inspired hydrogel, Ginsenoside Rh1, perianal ulcer, wound healing

## Abstract

Perianal ulcers remain challenging to treat because of persistent moisture, high mobility, recurrent infection, and prolonged inflammation at the lesion site. Herein, we developed a mussel-inspired, paintable hydrogel dressing loaded with ginsenoside Rh1 for multifunctional perianal ulcer management. The hydrogel was constructed from dopamine, chitosan, graphene oxide (GO), and polyvinyl alcohol, forming a physically and chemically dual-crosslinked network with robust wet-tissue adhesion, shear-thinning behavior, and structural stability. Optimization of the GO content revealed that the formulation with 0.5 mg/mL GO exhibited the best overall balance of mechanical properties, drug release behavior, and biocompatibility. The hydrogel showed pH-responsive sustained release of Rh1, low hemolysis (<2.45%), good cytocompatibility, antioxidant activity, and rapid hemostatic capability. Under 808 nm near-infrared irradiation, the GO-containing hydrogel also displayed stable photothermal responsiveness and effective antibacterial activity against Escherichia coli and Staphylococcus aureus. In a rat perianal ulcer model, the Rh1-loaded hydrogel significantly accelerated wound repair, achieving a healing rate of 96.78% by day 14. Histological analyses further confirmed enhanced re-epithelialisation, collagen deposition, and tissue remodeling after treatment. These results indicate that the DA/CS/GO/PVA-Rh1 hydrogel is a promising multifunctional dressing for the treatment of difficult-to-heal perianal ulcers.

## 1. Introduction

Perianal ulcers, characterized by their unique anatomical location, are prone to infection, suffer from high tissue tension, and exhibit significant mobility [1]. They are often accompanied by exudation, infection, and persistent inflammation, heal slowly, and are prone to recurrence [2,3]. Traditional dressings are difficult to secure, provide poor local barrier function, cause significant pain during dressing changes, and increase the patient’s psychological burden [4,5]. Clinical routine topical medications often only provide anti-inflammatory or anti-infection effects, making it difficult to simultaneously meet the needs for local adhesion, long-lasting drug release, and promotion of tissue regeneration [6,7,8]. Recent studies have shown that the complex local wound environment, bacterial infection, and oxidative stress can impair wound healing, highlighting the need for multifunctional dressings with stable adhesion, antibacterial activity, and microenvironment regulation [9,10]. Constructing a biomimetic, spreadable hydrogel dressing that can stably adhere to the complex perianal wound surface while providing antibacterial, hemostatic, localized drug delivery, and microenvironment-regulating functions is therefore of considerable significance for improving perianal ulcer treatment [11,12,13].

Dopamine (DA) is a typical mussel-inspired adhesive monomer that can self-polymerize into polydopamine (PDA). Its abundant catechol/quinone groups can achieve strong adhesion and reversible cross-linking at wet interfaces through various covalent and non-covalent interactions, providing a basis for adhesion and dynamic networks in hydrogels [14,15,16]. Chitosan (CS) has excellent biocompatibility, possesses both hemostatic and antibacterial properties, and can form multiple cross-linked networks with PDA, graphene oxide (GO), and polyvinyl alcohol (PVA), enhancing the mechanical and interfacial adhesion properties of hydrogels [17,18,19]. GO, as a two-dimensional nanomaterial, can enhance adhesion performance and near-infrared photothermal antibacterial effects, and through π-π stacking and hydrophobic interactions, it can synergistically carry drugs, improving the sustained release capability of ginsenoside Rh1 [20,21]. Ginsenoside Rh1, a bioactive ginseng-derived saponin, possesses prominent anti-inflammatory and antioxidant activities, which are beneficial for alleviating the adverse wound microenvironment [22,23,24]. In addition, Rh1 can promote tissue repair and regeneration, making it a promising therapeutic agent for accelerating ulcer healing when incorporated into a localized hydrogel delivery system. PVA, as a flexible framework, imparts spreadability and shear-thinning properties to the hydrogel, allowing it to closely adhere to irregular wound surfaces [25]. Multicomponent synergy enables the DA/CS/GO/PVA composite hydrogel to possess multiple functions, such as antibacterial properties, hemostasis, antioxidant effects, drug release control, and promotion of healing.

This study uses mussel foot silk as a biomimetic model to construct a DA/CS/GO/PVA stimuli-responsive hydrogel loaded with Rh1 for the treatment of perianal ulcers and conducts systematic structural design, performance regulation, and biological evaluation. By adjusting the GO content to optimize the physicochemical properties, the study investigates the drug release, hemostasis, antioxidant, antibacterial, and biocompatibility of the drug delivery system and evaluates its ability to regulate the wound microenvironment. Establish animal models to compare indicators such as wound healing and collagen remodeling, revealing their mechanisms and advantages in complex wound repair. This study can provide design ideas and experimental evidence for novel biomimetic hydrogels for difficult-to-heal wounds such as perianal lesions.

## 2. Experimental Section

### 2.1. Materials

Graphene oxide (GO) was purchased from Suzhou Carbon Source Graphene Technology Co., Ltd. (Suzhou, China). Chitosan (CS, MW = 50,000 Da) and polyvinyl alcohol (PVA, MW = 130,000 Da) were purchased from Sigma-Aldrich (St. Louis, MO, USA), Dopamine (DA), acetic acid (HAc), sodium hydroxide (NaOH), and formalin solution (NFB) were purchased from Aladdin (Shanghai, China). Kangfuxin solution was purchased from Sichuan Good Doctor Pharmaceutical Group Co., Ltd. (Chengdu, China). Ginsenoside (Rh1), phosphate-buffered saline (PBS), and phosphomolybdic acid (PMA) were purchased from Shanghai Macklin Biochemical Co., Ltd. (Shanghai, China). DMEM complete culture medium and CCK-8 detection kit were purchased from Beyotime Biotechnology (Shanghai, China). Sodium citrate anticoagulated whole blood (PRP) was purchased from Shanghai Yuchun Biotechnology Co., Ltd. (Shanghai, China). Penicillin–streptomycin solution (P-S solution) was purchased from HyClone (Logan, UT, USA). Mouse preosteoblasts (MC3T3-E1) were purchased from Procell Life Science & Technology Co., Ltd. (Wuhan, China). Deionized water was used throughout the experiment. Male Wistar rats were purchased from Changchun Yisi Experimental Technology Co., Ltd. (Changchun, China). All chemical reagents were used directly without purification.

### 2.2. Preparation of DA/CS/GO/PVA Hydrogel

CS was selected as the primary polymer component of the hydrogel dressing due to its excellent biocompatibility, hemostatic capability, and intrinsic antibacterial activity, and was dissolved in 1% (*v*/*v*) acetic acid to prepare a homogeneous precursor solution at a concentration of 10 mg/mL [17]. DA was selected as the mussel-inspired adhesive component due to its catechol-mediated wet-tissue adhesion, and 30 mg DA was dissolved in 10 mM HCl and activated with EDC/NHS to form amide bonds with the amino groups of CS [14]. After mixing into the CS solution, the DA loading corresponded to approximately 0.6 mg/mL in the total precursor volume, which is consistent with previously reported dopamine/chitosan systems that form homogeneous systems, where DA concentrations of 0.5–2.0 mg/mL and chitosan-type polymers at 10–20 mg/mL were used [26,27]. PVA was added to the DA/CS precursor to impart shear-thinning and spreadability for application onto moist, irregular perianal wounds. Graphene oxide (GO) was dispersed in deionized water and incorporated at 0, 0.2, 0.5, and 1 mg/mL to obtain the 0 GO, 0.2 GO, 0.5 GO, and 1 GO groups. The mixtures were stirred uniformly, adjusted to pH ≈ 7.4, degassed, cast into molds, and subjected to one freeze–thaw cycle (−20 °C for 12 h, room temperature for 6 h) to form the dual-crosslinked DA/CS/GO/PVA hydrogels through EDC/NHS-mediated chemical crosslinking and physical interactions. Among the four formulations, the 0.5 mg/mL GO group exhibited the best overall performance and was selected for Rh1 loading.

**Figure 1:**
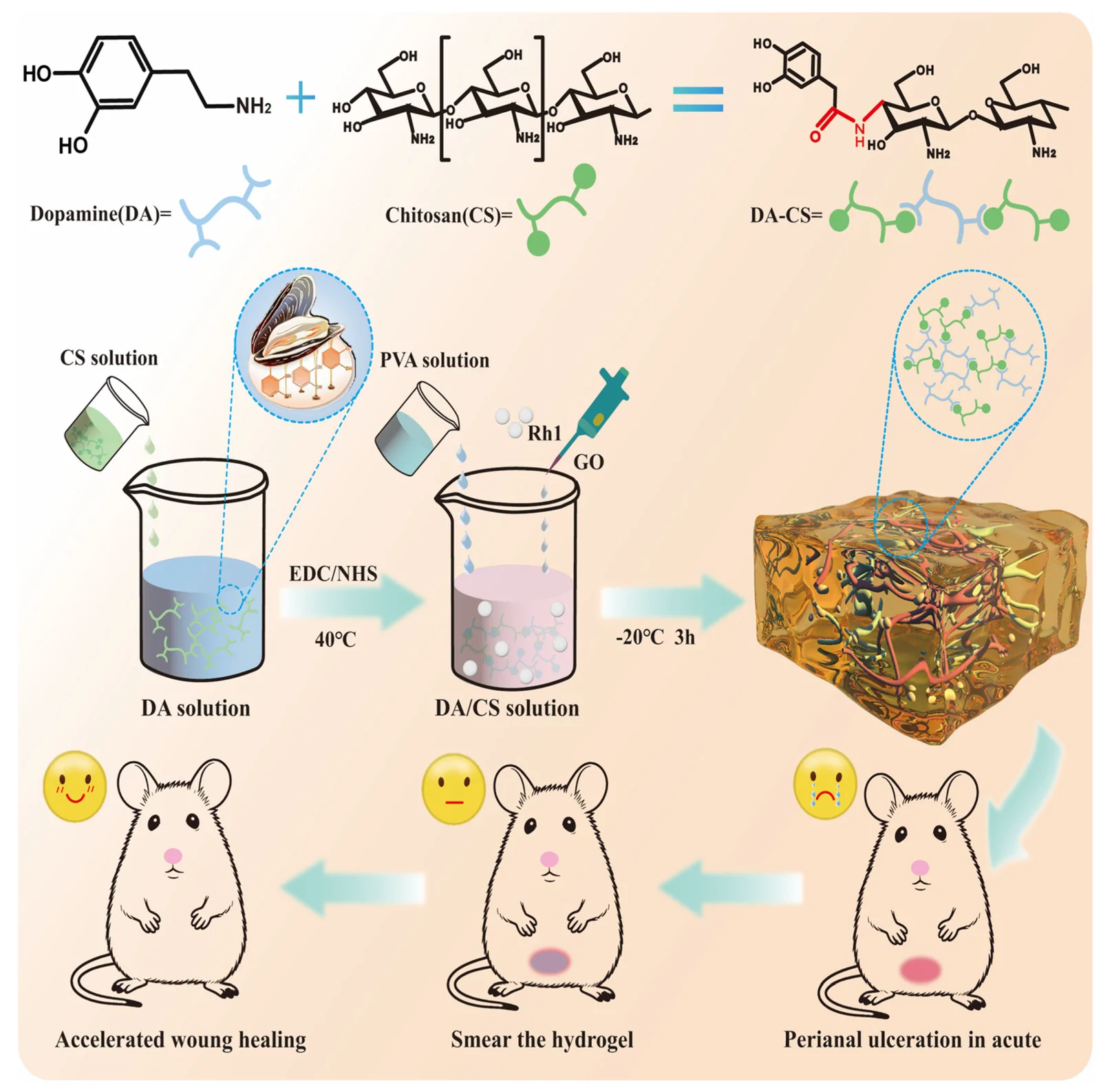
Flowchart for the Preparation of Bionic Stimulus-Responsive Paintable Hydrogel Dressings.

For the drug-loaded hydrogel (H-Rh1), ginsenoside Rh1 was dissolved in a small volume of aqueous ethanol, mixed into the 0.5 GO precursor before gelation, and subjected to the same pH adjustment, degassing, casting, and freeze–thaw treatment to obtain DA/CS/GO/PVA–Rh1 hydrogels The schematic illustration of the hydrogel fabrication process is presented in Figure 1.

### 2.3. Physicochemical Characterization of DA/CS/GO/PVA Hydrogels

#### 2.3.1. Macro and Micro Characterization

The gelation process and macroscopic morphology of the hydrogel dressings were visually recorded through image capture: the liquid precursor was injected into a transparent glass bottle, and its flow state was captured at an angle; after gelation, the same angle was used to capture the transition from sol to self-supporting gel for comparison. Using a scanning electron microscope (SEM) to observe the microstructure, the four groups of hydrogels were frozen in liquid nitrogen for 10 min, freeze-dried for 24 h, and then gold-coated before being observed at different magnifications.

#### 2.3.2. Fourier Transform Infrared Spectroscopy (FT-IR)

FT-IR spectroscopy was used to characterize the cross-linking effect of the DA/CS/GO/PVA hydrogel. After freeze-drying, the samples were ground and pressed with KBr, scanned in the range of 4000~500 cm^−1^, with a resolution of 4 cm^−1^, and scanned 32 times.

#### 2.3.3. Differential Scanning Calorimetry (DSC) and Thermogravimetric Analysis (TGA)

Differential scanning calorimetry and thermogravimetric analysis are used to characterize the thermal behavior and thermal stability of the hydrogel. DSC testing involves placing a 10 mg sample in a sealed aluminum crucible, with an empty crucible as a reference. The sample is first cooled to −50 °C and held at that temperature for 5 min to eliminate thermal history, then heated at a rate of 10 °C/min to 150 °C, with a continuous purge of high-purity nitrogen at 50 mL/min. For TGA analysis, approximately 14 mg of each sample was placed in an alumina crucible and heated to 280 °C at a rate of 10 °C/min under a nitrogen atmosphere, while the mass change was recorded. DTG curves were derived from the TGA data, from which T_onset_ and T_max_ were determined, and the residual mass was recorded at 280 °C.

#### 2.3.4. Rheological Properties

The rheological properties of four groups of hydrogels with different GO contents were tested using a rheometer. Conduct shear rate–viscosity scanning tests within the shear rate range of 0.1-100 s-1 to evaluate the shear-thinning properties of the four groups of hydrogels; fix the strain at 1%, set the test frequency range to 0.1–100 rad/s to explore the structural stability of the hydrogels under shear; fix the frequency at 1 rad·s−1, set the strain range to 1–500% to conduct strain scanning tests on the hydrogels, obtaining the changes in the storage modulus (G’) and loss modulus (G″) of the four groups of hydrogels with strain.

#### 2.3.5. In Vitro Degradation Performance

The in vitro degradation performance of the four types of smearable hydrogels was evaluated using the mass loss method. Weigh 0.3 g and record it as the initial mass M_0_, then immerse it in centrifuge tubes containing PBS buffer solutions with different pH values (pH = 5.4/7.4/8.4), and incubate at 37 °C with constant shaking to simulate physiological conditions. Samples were taken at 2, 4, 6, 8, 10, 12, 24, and 48 h, and the surface moisture was absorbed with filter paper before weighing and recorded as M_t_. The degradation degree was calculated according to Equation (1), and the corresponding degradation curves were plotted to evaluate the degradation behavior of the samples.

(1) $$\mathrm{Degradation rate}\left(\%\right)=\frac{M_{0}-M_{t}}{M_{t}}\times 100\%$$

#### 2.3.6. Adhesion Performance

(1) Apparent adhesion of the mouse heart, liver, spleen, lung, and kidney:

Taking the 0.5 GO group hydrogel as an example, evenly spread a certain amount of the hydrogel sample on a glass slide, tightly adhere the gel surface to the surface of the ex vivo tissue, apply constant pressure for 30 s to ensure full contact and then lift the slide to observe the adhesion of each group of hydrogel samples.

(2) Overlap Shear Test of Pigskin:

After processing, cut the fresh pigskin into 15 mm × 40 mm samples, dry them, and apply hydrogel in the 10 mm × 10 mm overlap area. Then, place the two pieces of pigskin in an overlapped position and apply constant light pressure. Clamp the sample in an electronic universal testing machine (1 m/s), record the load–displacement curve, and calculate the maximum shear strength using the formula.

### 2.4. In Vitro Drug Release Characterization of DA/CS/GO/PVA Hydrogels

By simulating the pH value changes in the microenvironment of the lesion during critical healing periods, the drug release behavior of drug-loaded hydrogel dressings at different stages of skin wound healing was investigated. Phosphate buffer solutions with pH values of 5.4, 7.4, and 8.4 were precisely prepared to simulate the typical physiological and biochemical environments of the inflammatory phase, proliferation phase, and remodeling phase, respectively.

Prepare gradient concentrations of ginsenoside Rh1 solution, measure the absorbance at a wavelength of 203 nm using a UV spectrophotometer, and establish a drug standard curve. Weigh 3 g of drug-loaded hydrogel dressings with different GO contents (0 GO/0.2 GO/0.5 GO/1 GO), place them in dialysis bags, add 2 mL of phosphate solution, seal them, and then place them in sealed bottles containing 50 mL of phosphate-buffered solution. Finally, put them in a constant-temperature shaker. At different time points, 5 mL of the solution is withdrawn, and an equal volume of buffer is added. The absorbance is measured and substituted into the standard curve to calculate the concentration. The cumulative release rates of drugs in vitro for the four groups of samples within 72 h were analyzed. The cumulative drug release was determined as described in Equation (2). Here, V_t_ represents the total volume of the release medium; C_t_ and C_i_ are the drug concentrations at the t-th and i-th time points, respectively; V_i_ is the sample volume for each sampling; and M_0_ is the total drug loading of the hydrogel.

(2) $$\mathrm{Cumulative drug release rate}\left(\%\right)=\frac{V_{t}\times C_{t}+V_{i}\times \sum_{n=1}^{t-1}C_{i}}{M_{0}}\times 100\%$$

### 2.5. In Vitro Biological Characterization of DA/CS/GO/PVA Hydrogels

#### 2.5.1. Cytotoxicity Test

The CCK8 method was used to assess the effective biocompatibility of the composite gel, measuring the activity and proliferation of MC3T3-E1 cells at a wavelength of 450 nm.

#### 2.5.2. Hemolysis Test

Biomedical materials need to possess low or no hemolytic properties. The hemolysis test was used to evaluate their blood compatibility and impact on red blood cells. This experiment uses four groups of hydrogels with different GO content to evaluate their hemolytic response and verify their safety as wound dressings. Fresh rabbit blood was washed with saline to prepare a 2% red blood cell suspension; 0.2 g of the hydrogel sample was extracted in saline at 37 °C for 2 h, then mixed with the red blood cell suspension and incubated in a water bath at 37 °C for 4 h. After centrifugation, take the supernatant and measure its absorbance at 540 nm. The hemolysis rate was calculated according to Equation (3). Set up 3 parallel samples for each group.

(3) $$\mathrm{Hemolysis rate}\left(\%\right)=\frac{{\mathrm{OD}}_{\mathrm{sample}}-{\mathrm{OD}}_{\mathrm{NaCl}}}{{\mathrm{OD}}_{H_{2}O}-{\mathrm{OD}}_{\mathrm{NaCl}}}\times 100\%$$

#### 2.5.3. Antioxidant Experiment

The antioxidant capacity of the biomimetic hydrogel dressing was evaluated using the DPPH radical scavenging method. DPPH solution was mixed with the extract of hydrogel samples containing different GO contents from four groups. Three sets were established: experimental (A_s_), control (A_c_), and blank (A_b_). The absorbance at 517 nm was measured using a microplate reader, and the UV absorption spectrum was analyzed using a UV spectrophotometer. The antioxidant rate of the composite hydrogels with different graphene oxide contents was calculated according to Equation (4).

(4) $$\mathrm{DPPH scavenging rate}\left(\%\right)=\left(1-\frac{A_{s}-A_{c}}{A_{b}}\right)\times 100\%$$

#### 2.5.4. Mouse Tail Amputation Hemostasis Experiment

Mice were randomly divided into four groups (n = 3 per group): the blank group (Blank), gauze group (Gauze), drug-free hydrogel group (Hydrogel), and drug-loaded hydrogel group (H-Rh1). All animal experiments were approved by the Animal Experiment Ethics and Welfare Review Committee of the Basic Medical College of Jilin University and conducted in accordance with the ARRIVE guidelines for reporting animal research. All hydrogels used were from the 0.5 GO group. After anesthesia with isoflurane, the tail of the mouse was amputated about 3 mm from the tip, and the timer was started. For the three groups other than the blank group, the samples were gently pressed or applied to the wound and left for 1 min. Once the mouse tail is completely hemostatic, quickly weigh the blood-soaked filter paper and record the value. The total blood loss is calculated by subtracting the dry weight of the filter paper from its wet weight.

#### 2.5.5. Coagulation Experiment

The in vitro whole blood coagulation index (BCI) method was used to evaluate the pro-coagulation performance of DA/CS/GO/PVA Rh1 drug-loaded hydrogel dressings. The experiment was set up with a blank group, a gauze group, a 0 GO group, a 0.2 GO group, a 0.5 GO group, a 1 GO group, and a 0.5 GO drug-loaded group (H-Rh1), totaling 7 groups. Prepare the hydrogels for each group into circular disks with a diameter of 10 mm and a thickness of 2 mm, and preheat at 37 °C for later use. Take 50 μL of sodium citrate-anticoagulated fresh whole blood from mice, add 5 μL of 0.2 mol/L CaCl_2_ to activate it, then drop it onto the surface of the sample, and incubate for 10 min; subsequently, add 5 mL of deionized water to rinse off the unclotted blood cells, let it stand or centrifuge, then collect the supernatant and measure the absorbance at a wavelength of 540 nm. The blood coagulation index (BCI) of different samples was calculated according to Equation (5), where the sample group is denoted as A_s_, deionized water as A_w_, and the untreated 50 μL whole blood as A_n_ (negative control).

(5) $$\mathrm{BCI}\left(\%\right)=\frac{A_{s}-A_{w}}{A_{n}-A_{w}}\times 100\%$$

#### 2.5.6. Photothermal Antibacterial Experiment

(1) Near-Infrared “Photothermal Switch”

Effectively prepare hydrogels into 11 mm diameter, 2 mm thick blocks and place them in a 48-well plate. Irradiate with NIR at 808 nm for 10 min, removing the laser every 1 min and recording the surface temperature. At the optimal power density, perform a cycle of 10 min of irradiation and 10 min of removal on the 0.5 GO group hydrogel three times, recording the temperature to investigate photothermal stability.

(2) Antibacterial Performance of NIR-Responsive Hydrogels

To assess the antibacterial properties of the biomimetic hydrogel dressing against Escherichia coli and Staphylococcus aureus, the bacterial strains were cultured to the exponential growth phase. The hydrogel was then treated with the bacterial suspension and subjected to 10 min of NIR irradiation at 808 nm and 2 W/cm^2^, followed by a 24-h co-culture. After resuspension and coating with PBS, the bacterial colonies were counted, and the antibacterial rate was calculated using the formula in Equation (6). Additionally, the bacterial morphology and quantity were observed using scanning electron microscopy after sample treatment. Where C_s_ and C_con_ represent the colony counts of the hydrogel sample group and the blank control group, respectively.

(6) $$\mathrm{Antibacterial rate}\left(\%\right)=\frac{C_{s}-C_{\mathrm{con}}}{C_{\mathrm{con}}}\times 100\%$$

### 2.6. Animal Wound Healing Test of DA/CS/GO/PVA Hydrogel

#### 2.6.1. Experimental Animals

Twenty-five male Wistar rats aged 8-9 weeks and weighing 200–250 g were randomly assigned to five groups (n = 5 per group): Blank control group (Blank), Hydrogel group (Hydrogel), Rh1 group (Rh1), Positive control group (Positive), and H-Rh1 group. All animal experiments were approved by the Animal Experiment Ethics and Welfare Review Committee of the Basic Medical College of Jilin University and performed in accordance with the ARRIVE guidelines for reporting animal research.

#### 2.6.2. Construction of Skin Ulcer Wound Model

After fasting for 24 h, rats were anesthetized and fixed by intraperitoneal injection of 3% pentobarbital sodium, and the perianal skin was disinfected. Ice acetic acid (1.5–2.5 mL per point, 75%) was subcutaneously injected at 3, 6, and 9 o’clock positions around the anus. After 48 h, perianal ulcers with redness and inflammatory exudate were observed, indicating successful modeling.

#### 2.6.3. Intervention Measures

Blank Control Group (Blank): Model established only; Hydrogel Group (Hydrogel): 0.5 GO group with hydrogel without drugs evenly covering the ulcer surface and undergoing NIR irradiation (10 min); Ginsenoside Rh1 Raw Drug Group (Rh1): Rh1 solution (0.5 mL, 10 mg/mL) evenly dripped onto the ulcer surface; Positive Control Group (Positive): Kangfuxin Liquid used to rinse the ulcer surface; 0.5 GO Hydrogel Loaded with Rh1 Drug Group (H-Rh1): a DA/CS/GO/PVA-Rh1 smearable hydrogel dressing evenly applied daily to the rat’s anal ulcer, also undergoing NIR irradiation (10 min). Each group received treatment once daily for 14 consecutive days, and the basic vital signs of the rats were recorded throughout the experimental period. On days 4, 7, and 14 after treatment, photographs were taken to monitor wound healing progression, and the wound area was measured to calculate the wound closure rate. Calculate the wound closure rate to evaluate the speed of wound healing by Equation (7), where A_0_ and A_t_ represent the wound area on day 0 and day t, respectively.

(7) $$\mathrm{Wound closure rate}\left(\%\right)=\frac{A_{0}-A_{t}}{A_{0}}\times 100\%$$

#### 2.6.4. H&E Staining

Take paraffin sections (5 μm) of skin ulcer tissue, perform routine xylene dewaxing and gradient ethanol hydration; stain nuclei with hematoxylin for 5 min, differentiate them with 1% hydrochloric acid alcohol for 3 s, and turn them blue by treating with 0.5% ammonia water for 5 min; stain cytoplasm with eosin for 2 min, dehydrate with gradient ethanol, clear with xylene, then mount with neutral gum. Observe the morphology of the wound tissue and measure skin thickness under an optical microscope.

#### 2.6.5. Masson Staining

Obtain paraffin sections (5 μm) of skin ulcer tissue, deparaffinize and hydrate, then stain with hematoxylin, acid fuchsin, and aniline blue in sequence. After dehydration and clearing, mount with neutral gum and observe collagen deposition under a light microscope.

### 2.7. Statistical Analysis

Unless otherwise stated, experiments were performed with three independent replicates. Data processing and visualization were performed using Origin software, and statistical analysis was conducted using one-way analysis of variance. A *p*-value of less than 0.05 was considered statistically significant.

## 3. Experimental Results and Discussion

### 3.1. Basic Characterization of DA/CS/GO/PVA Hydrogels

#### 3.1.1. Macro and Micro Morphology

**Figure 2:**
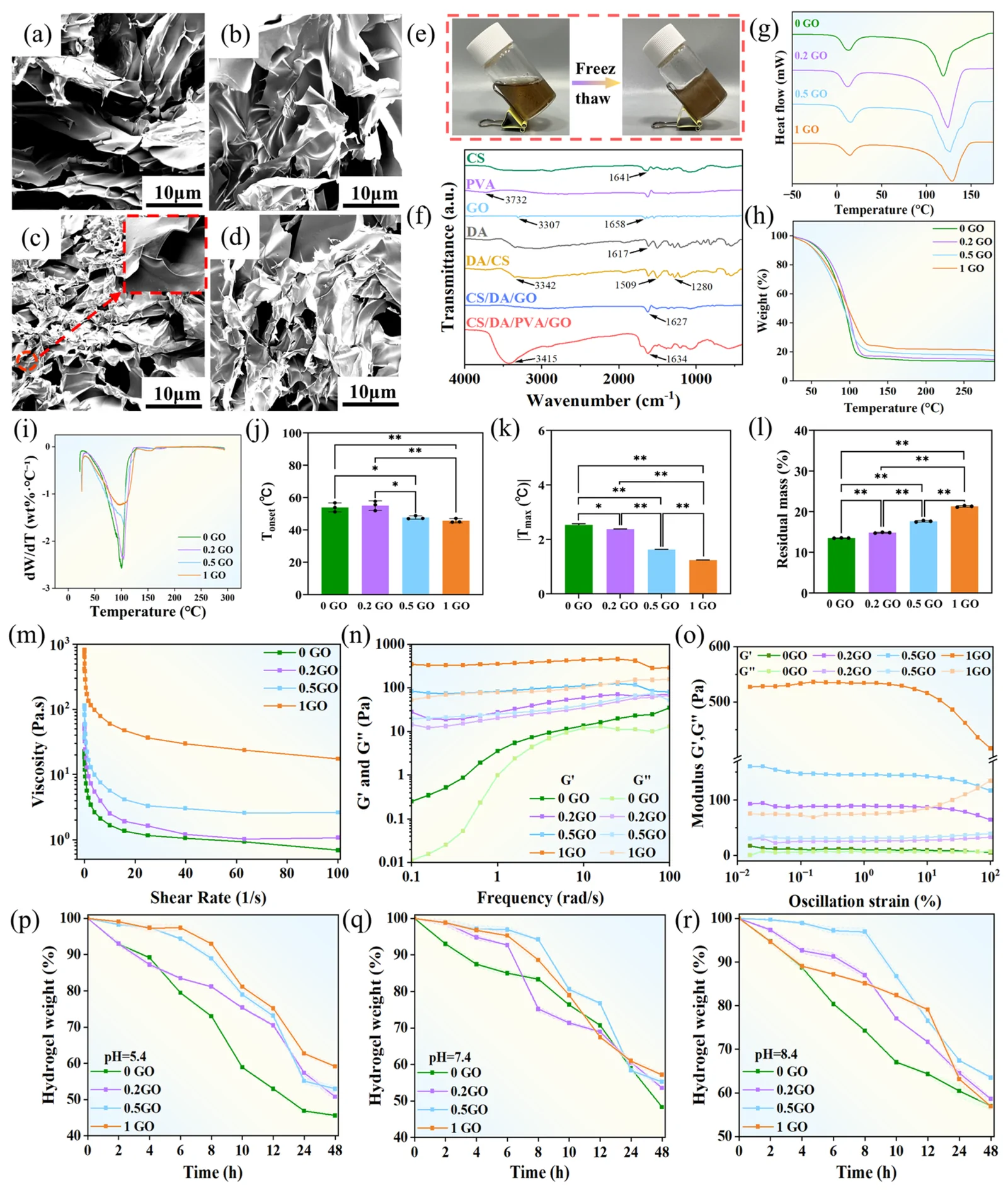
Basic characterization of hydrogels: (**a**–**d**) SEM images of different GO contents, namely 0 GO, 0.2 GO, 0.5 GO, and 1 GO; (**e**) macroscopic sol–gel state; (**f**) infrared spectrum of smearable hydrogel; (**g**) differential scanning calorimetry; thermal stability analysis: (**h**) thermogravimetric analysis; (**i**) derivative thermogravimetric; (**j**) onset degradation temperature; (**k**) maximum degradation temperature; (**l**) residual mass; rheological properties: (**m**) shear thinning, (**n**) frequency sweep, (**o**) strain sweep; hydrogel degradation performance: (**p**) pH = 5.4, (**q**) pH = 7.4, (**r**) pH = 8.4.

The micro-porous structure of hydrogels with different graphene oxide (GO) contents was observed using SEM (Figure 2a–d). The results showed that all four groups of samples formed a typical three-dimensional porous network structure. This characteristic is attributed to the “ice crystal template effect” during the freeze–thaw process of polyvinyl alcohol (PVA): during the freezing stage, water molecules crystallize to form ice crystals, and the components of the hydrogel are enriched in the gaps between the ice crystals. During the thawing stage, the sublimation or melting of the ice crystals leaves through-holes, and the concentrated polymer molecular chains form stable pore walls through hydrogen bonding and electrostatic interactions, constructing a porous layered framework [28].

As shown in Figure 2e, the hydrogel precursor solution maintains a parallel liquid level with the experimental table surface inside the glass bottle. After 3 h of freeze–thaw treatment, it can still maintain morphological stability and remain parallel to the bottom of the container on the inclined wall, indicating that the system has successfully transformed from a solution state to a gel state. This confirms that the network constructed by freeze–thaw physical cross-linking has good self-supporting properties, and the hydrogel samples are prepared satisfactorily.

The introduction of GO significantly optimizes the microstructure of the hydrogel matrix: samples with low GO content (Figure 2a) have a loose pore structure and smooth pore walls; as the GO content increases, the pore walls gradually become rougher and exhibit a wrinkled, layered structure, indicating that the two-dimensional nanofiller GO has successfully embedded into the PVA/CS/DA crosslinked network, making the network structure denser and more complex. An appropriate amount of GO (Figure 2b,c) can retain the interconnected pore structure, facilitating the adsorption and swelling of exudates and the diffusion of drugs, thereby laying a structural foundation for subsequent performance studies. However, excessive GO (Figure 2d), while further enhancing compactness, may reduce liquid permeability and increase material brittleness.

#### 3.1.2. Fourier Transform Infrared Spectroscopy

Figure 2f shows the FTIR spectra of the DA/CS/GO/PVA composite hydrogels, which were used to investigate the interactions among different components. The characteristic peaks of CS and PVA were observed at 1641 cm^−1^ (amide I band, C=O stretching) and 3732 cm^−1^ (O–H stretching vibration), respectively. For the DA/CS system, the characteristic bands at 1509 and 1280 cm^−1^ were associated with the oxidation and polymerization of dopamine. Meanwhile, the EDC/NHS-mediated activation of dopamine carboxyl groups facilitated the covalent coupling between dopamine and amino groups of chitosan, resulting in the formation of amide bonds and contributing to the chemical crosslinking of the hydrogel network [29]. After incorporation of GO, the O–H stretching band broadened and shifted to 3415 cm^−1^, accompanied by changes in the C=O vibration region (1627–1634 cm^−1^), indicating enhanced hydrogen bonding interactions among GO, PVA, CS, and DA. These results demonstrate that the DA/CS/GO/PVA hydrogel network was stabilized through the synergistic effects of chemical crosslinking and physical interactions [21].

#### 3.1.3. Differential Scanning Calorimetry (DSC) and Thermogravimetric Analysis (TGA)

Figure 2g,h show the DSC and TGA curves of DA/CS/GO/PVA composite hydrogels with different GO contents, respectively. The DSC curves show that all four samples exhibit endothermic peaks at 100–150 °C, corresponding to the endothermic processes of water evaporation and the breaking of physical cross-linking bonds. The peak temperature of the hydrogel without GO is the lowest, and as the GO content increases, the endothermic peak shifts to a higher temperature, indicating that GO significantly enhances the thermal stability of the hydrogel. This is attributed to the strong hydrogen bonding interactions formed between the oxygen-containing functional groups (-COOH, -OH) on the surface of GO and the -OH of PVA, the -NH_2_ of CS, and the phenolic hydroxyl groups of PDA, which increase the physical cross-linking points, enhance the stability of the polymer network, and improve the cohesive energy density [30].

TGA and DTG analysis were performed to quantitatively evaluate the thermal behavior of the GO-containing hydrogels. The TGA curves reveal that the thermal mass-loss process can be divided into two primary stages. The prominent weight loss event below 120 °C was mainly attributed to the evaporation of free and bound water trapped within hydrogel networks, rather than the backbone decomposition of the polymer matrix. The second stage ranging from 120–250 °C corresponds to the gradual release of strongly bound water and intrachain dehydration of polymer chains. DTG curves (Figure 2i) were applied to determine T_max_, which represents the temperature at the maximum mass loss rate during water evaporation. The position and intensity of DTG peaks varied with GO loading. Quantitative statistical (Figure 2j,k) results showed that the neat hydrogel (0 GO) exhibited high T_onset_ (~54 °C, extracted from TGA) and T_max_ (extracted from DTG), while the 1 GO sample displayed low values of these two parameters, with fluctuations observed among intermediate GO-loaded groups. This variation indicates that the introduction of GO alters the hydrogen-bond network of the hydrogel, modulating the binding strength between polymer chains and water molecules. In contrast, Figure 2l shows that the residual mass at 280 °C monotonically increased from ~13.5 wt.% (0 GO) to ~21.2 wt.% (1 GO) with rising GO concentration. This phenomenon originates from the high thermal stability of GO nanosheets. The incorporated GO participates in interfacial hydrogen bonding within the hydrogel network, which facilitates the formation of carbonized residue during heating. One-way ANOVA with Tukey’s post-hoc test demonstrates statistically significant intergroup differences for all three thermal parameters (* *p* < 0.05, ** *p* < 0.01). In summary, the incorporation of GO modulates the water-binding state of hydrogel networks and elevates the final carbon residue, which is beneficial for improving the thermal resistance of the smearable hydrogel [31].

#### 3.1.4. Rheological Properties

(1) Shear Thinning

The apparent viscosity of DA/CS/GO/PVA composite hydrogels with different GO contents varies with shear rate, as shown in Figure 2m. All four groups of samples exhibit typical shear-thinning behavior of non-Newtonian fluids within the shear rate range of 1–100 s^−1^, with the apparent viscosity significantly decreasing as the shear rate increases. This phenomenon is attributed to the dynamic destruction and reconstruction of the hydrogel’s three-dimensional network under shear force: at low shear rates, the polymer molecular chains and GO sheets form intact physical cross-linking points through hydrogen bonding, causing the molecular chains to intertwine and the hydrogel to maintain a higher viscosity; as the shear rate increases, the shear force disrupts these temporary physical cross-linking points, which macroscopically manifests as a decrease in viscosity. At the same shear rate, the initial viscosity and the degree of decrease of the hydrogel increase with the GO content, indicating that GO can serve as an effective physical cross-linking point, significantly enhancing the density and strength of the hydrogel network, making its structure more stable in a stationary state, and endowing the material with excellent spreadability [32].

(2) Frequency Mode

Figure 2n shows the frequency scan curve of the dynamic shear of the hydrogel, which can reflect its viscoelastic response. In the range of 0.1~100 rad·s^−1^, the storage modulus (G’) of all four groups of samples is consistently higher than the loss modulus (G″), exhibiting typical elastic solid characteristics; as the GO content increases, G’ and G″ both increase overall across the entire frequency range, indicating that the introduction of GO effectively enhances the mechanical strength of the hydrogel. In addition, the G’ and G″ of all samples remain stable in the high-frequency region, and no crossover point where G″ exceeds G’ is observed. This is due to the strong hydrogen bonding formed between the oxygen-containing functional groups on the surface of GO and the main network of the hydrogel, confirming that its three-dimensional network structure has good stability [33].

(3) Strain Mode

The strain sweep curve (Figure 2o) is used to examine the nonlinear viscoelasticity and structural stability of the hydrogel network. In the low strain range, the G’ of the GO-containing samples is always higher than G″, indicating that their micro-network structure is stable. The G’ of the hydrogel without GO gradually decreases with increasing strain, while G″ continues to rise. After the strain exceeds the yield strain, G″ surpasses G’, and the hydrogel transitions from a gel state to a sol state. However, with the increase in GO content, the G’ in the linear viscoelastic region of the hydrogel significantly increases, and the critical yield strain also shows an increasing trend. This proves that GO not only enhances the stiffness of the hydrogel, but its two-dimensional layered structure can also effectively disperse and dissipate energy, improve the network’s resistance to large deformations, delay the yield limit, and endow the material with superior toughness [34].

#### 3.1.5. In Vitro Degradation Performance

Figure 2p shows that all hydrogels rapidly degraded within 48 h in an acidic environment (pH = 5.4), with the GO content significantly affecting their stability: the blank group without GO had the fastest degradation rate, with 54.15% degradation at 12 h and the lowest remaining weight at 48 h; as the GO content increased, the degradation curve shifted upward, and degradation was effectively delayed, with the 1 GO group showing only 41.88% degradation at 48 h. This is because the GO layers form a dense hydrogen bond network with the polymer chains, increasing the cross-linking density and enhancing the hydrogel’s resistance to acidic hydrolysis. In PBS solution at pH = 7.4 (Figure 2q), the degradation rate of GO-containing hydrogels is significantly slower than in acidic environments; the 12-h degradation rate of the 1 GO group (43.52%) is higher than that of the 0.5 GO group (24.27%). The remaining weight ratio of the 1 GO group after 48 h is still the highest, consistent with the results in acidic environments.

In a weakly alkaline environment (pH = 8.4) (Figure 2r), the hydrogel degradation is most severe, indicating that alkaline conditions are more corrosive to the network structure. However, the degradation rate of the GO-containing group is still significantly lower than that of the GO-free group, because the GO two-dimensional sheets form a physical barrier, hindering the penetration of OH^−^ and water molecules into the gel, thereby delaying the hydrolysis of the polymer chains. In summary, GO can comprehensively enhance the tolerance of hydrogels in different chemical environments. Its layered structure reduces the pore size of the hydrogel and makes the penetration path more tortuous, collectively endowing the hydrogel with superior structural integrity and resistance to degradation [35]. However, it should be noted that the current degradation evaluation was mainly based on pH-dependent conditions, which cannot fully reproduce the complex physiological environment of perianal wounds; therefore, future studies incorporating enzyme-mediated degradation models and long-term in vivo degradation assessments are necessary to further evaluate the degradation behavior and application potential of the hydrogel system.

### 3.2. Physicochemical Properties of DA/CS/GO/PVA Hydrogels

#### 3.2.1. Adhesive Properties

(1) Apparent Adhesion to Mouse Heart, Liver, Spleen, Lungs, and Kidneys

**Figure 3:**
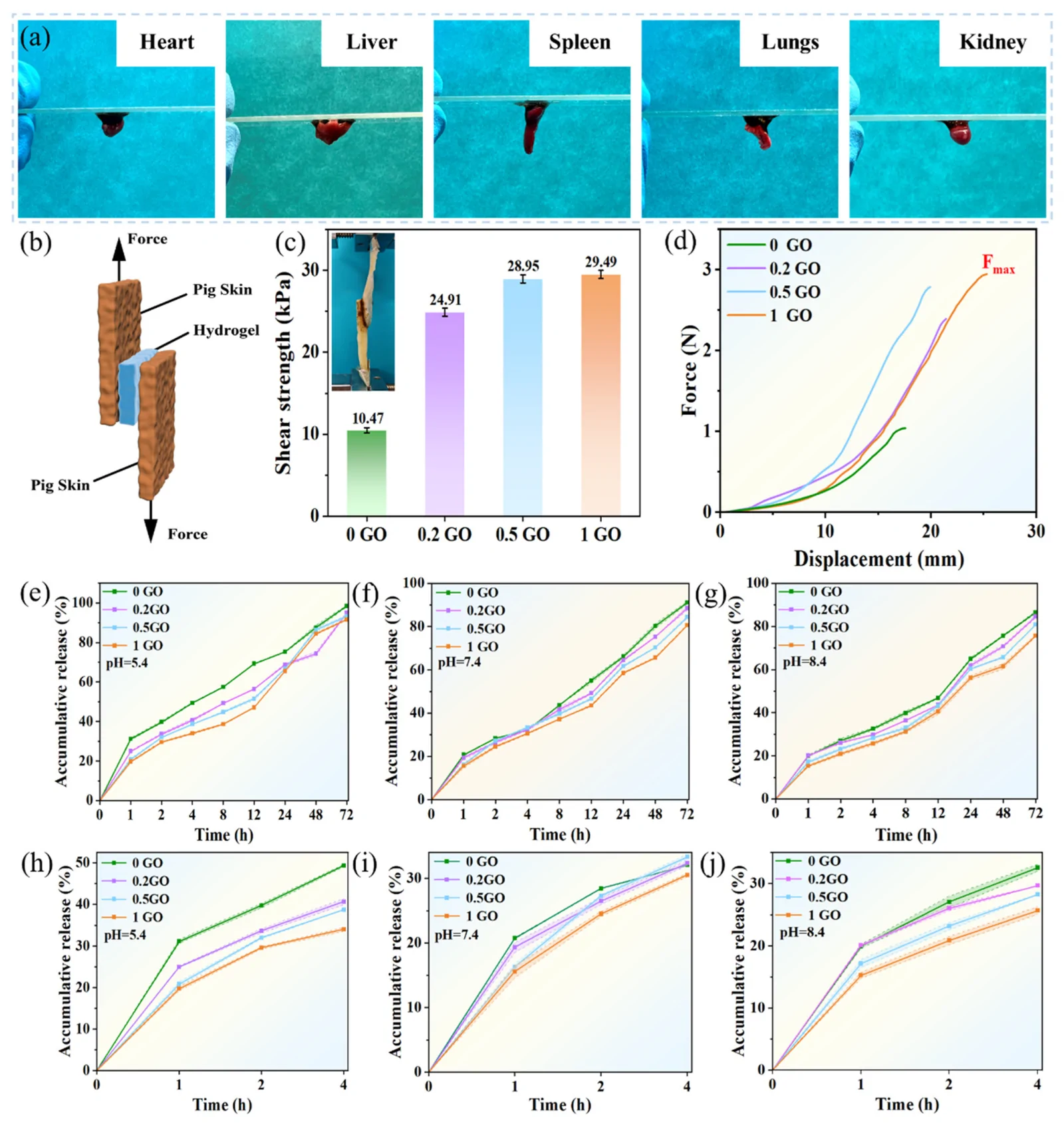
Adhesion performance of the smearable hydrogel on pig skin: (**a**) apparent adhesion of mouse organs, (**b**) schematic diagram of pig skin lap shear, (**c**) shear strength, (**d**) shear force–displacement curve; drug release performance of the smearable hydrogel dressing in PBS at different pH values (pH = 5.4/pH = 7.4/pH = 8.4): (**e**–**g**) drug release over 0–72 h, (**h**–**j**) drug release over 0–4 h.

Figure 3a visually demonstrates the apparent adhesion of 0.5 GO group smearable hydrogel samples to the wet, irregular, and tender surfaces of the mouse heart, liver, spleen, lungs, and kidneys. The hydrogels can firmly adhere to the moist, irregular, and tender surfaces of various organs without spontaneous detachment, which preliminarily proves that this series of hydrogels has a certain degree of tissue adhesion. Its adhesion mechanism is mainly attributed to the following synergistic effects: (1) The catechol groups contained in the PDA component of the hydrogel can form imine bonds or hydrogen bonds with amino groups and other groups on the tissue surface; (2) Electrostatic interactions occur between the cations of PDA and CS and the anions on the tissue surface; (3) The physical interpenetration of the hydrogel polymer network with the tissue surface and the hydrogen bonding also provide adhesion. The high adhesion ability provides the greatest assurance for the application of DA/CS/PVA/GO adhesive hydrogel as a biomedical dressing [36].

(2) Shear Test of Pigskin Overlap

As shown in Figure 3b,c, the adhesion strength of the hydrogel significantly increases with higher GO content. The shear strength of the 1 GO group reaches 29.49 kPa, which is 2.8 times that of the blank control group (10.47 kPa), confirming that GO can effectively enhance the tissue adhesion performance of the hydrogel. Its enhancement mechanism is mainly reflected in two aspects: first, GO has a large specific surface area and abundant oxygen-containing functional groups (COOH, OH), which can form multiple hydrogen bonds and mechanical interlocking with proteins and polysaccharides on the tissue surface, enhancing the interfacial adhesion; second, GO improves the mechanical strength and toughness of the hydrogel itself, making it less prone to cohesive failure under shear loads, thereby facilitating the effective transmission of stress to the adhesion interface [37]. Figure 3d shows the shear force–displacement curves of pigskin lap shear, indicating that the curves of all groups exhibit a nonlinear rise to the maximum force Fmax, followed by interface failure. The 0 GO group has the smallest slope, the shortest yield displacement, and the earliest debonding; after adding GO, the slope and peak force of the 0.2 GO group significantly increase; although the 0.5 GO group reaches Fmax (2.78 N) earlier, it is lower than the 1 GO group (2.95 N). The 1 GO group can still maintain the load at a larger displacement, indicating better adhesion stability. In comparison, the area under the curve of GO-modified hydrogels is larger, indicating higher adhesive toughness [38]. Moreover, the displacement required to reach the peak force increases with the GO content, suggesting that it can withstand greater deformation before interface failure, endowing the hydrogel with more stable and damage-resistant tissue adhesion effects.

#### 3.2.2. Analysis of Drug Release Performance of DA/CS/GO/PVA Hydrogel

Figure 3e–g show the cumulative drug release curves of the hydrogel in PBS solutions at pH = 5.4, 7.4, and 8.4 over 72 h. All groups exhibited a biphasic release characteristic with an initial rapid release followed by a gradual plateau. In an acidic environment (pH = 5.4), the drug release rate is the fastest, and the cumulative release amount is the highest, reaching a cumulative release rate of 66.68% at 24 h. Excessive GO tends to agglomerate, forming structural defects, which causes the cumulative release rate of the 1 GO group to rise to 84.36% at 48 h, with a release rate slightly faster than that of the 0.2 GO group. This is due to the protonation-induced swelling of the CS molecular chains and partial bond hydrolysis under acidic conditions. In neutral and alkaline environments, the hydrogel structure is more stable, and the drug release rate significantly slows down. GO has a notable regulatory effect on the drug release behavior; an appropriate amount of GO (0.5 GO, 1 GO group) can enhance network density and delay drug diffusion [39]. The cumulative drug release rates at 72 h are 80.86~84.37% and 75.7~80.67%, respectively.

Figure 3h–j focus on the initial release phase at 4 h, where all hydrogels exhibit a burst release phenomenon due to the rapid diffusion of the drug in the loose network. The drug release rate exceeds 60% in acidic conditions at 4 h, while the burst release effect is significantly alleviated in neutral and alkaline environments. The introduction of GO can effectively inhibit the initial burst release, with the drug release amount at 4 h in the 0.5 GO group being significantly lower than that in the 0 GO group, confirming that its dense network can delay the early diffusion of the drug and the degradation of the hydrogel matrix. This hydrogel releases the drug quickly in an acidic microenvironment and provides long-lasting and stable drug release under neutral physiological conditions. GO is a key regulatory factor, with the 0.5 GO group showing the best drug release effect. Both excess and insufficient GO can affect the release behavior of Rh1.

### 3.3. In Vitro Biological Analysis of DA/CS/GO/PVA Hydrogel

#### 3.3.1. Hemolysis Test

**Figure 4:**
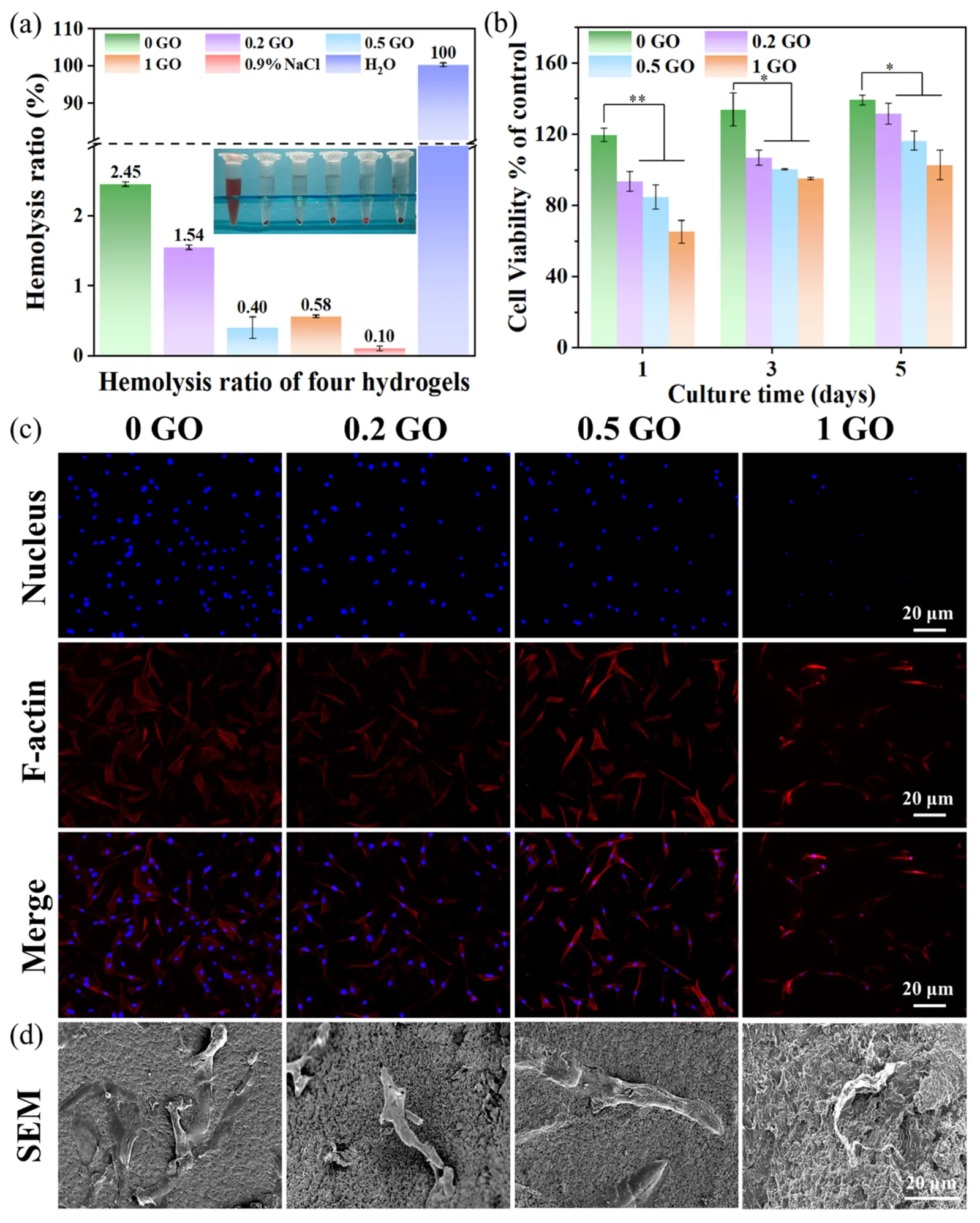
In vitro biocompatibility of hydrogels: (**a**) hemolysis rate; (**b**) relative cell proliferation rate; (**c**) cell staining images; (**d**) cell electron microscopy images. (* *p* < 0.05, ** *p* < 0.01).

The in vitro hemolytic performance of the DA/CS/GO/PVA composite hydrogel is shown in Figure 4a. According to international standards (such as ISO 10993-4), the hemolysis rate of the material should be less than 5% to be considered to have good blood compatibility. From the quantitative statistical results, it can be seen that the hemolysis rates of all experimental group hydrogels are far below this safety threshold; the hemolysis rate of the negative control (0.9% NaCl solution) is extremely low at approximately 0.1%, while the positive control (H_2_O) caused nearly 100% complete hemolysis, demonstrating the effectiveness of the experimental system. More importantly, the hemolysis rates of all hydrogel samples are controlled at an extremely low level below 2.45%, and with the increase in GO content, the hemolysis rates do not show a significant upward trend, proving that the introduction of GO does not induce additional hemolytic risk, and the material itself is safe for red blood cell membranes. Figure 4b shows the hemolytic effects of each sample, providing visual evidence for the aforementioned quantitative data. Among them, the liquid in the positive control group (H_2_O) is bright red and completely transparent, indicating that the red blood cells have completely ruptured and hemoglobin has fully released into the solution. In contrast, the solution color in all the hydrogel extract tubes is close to that of the negative control group (0.9% NaCl), remaining clear and transparent with only a very faint red hue. This indicates that only a small amount of hemoglobin has leaked from the red blood cells, perfectly matching the quantitative data in Figure 4a. In summary, DA/CS/GO/PVA composite hydrogels with different GO contents all exhibited excellent blood compatibility, showing no destructive effects on red blood cell membranes, thus meeting the safety requirements for biomedical materials in direct contact with human blood.

#### 3.3.2. CCK-8 Method for Detecting Cell Proliferation

As shown in Figure 4b, except for the 1 GO group, the cell viability of the other three sample groups was above 80% at 1, 3, and 5 days of culture, demonstrating good biocompatibility. On the first day of culture, the cell viability in the 0 GO and 0.2 GO groups was similar (92~100%), while the 1 GO group showed a relatively lower viability of approximately 65.19%, indicating that high-concentration GO may exert an initial inhibitory effect on cell attachment and early proliferation.

As the culture time extended to 3 and 5 days, the cells in each group showed a normal proliferation trend, confirming that the hydrogel has good cell compatibility. The GO content has a concentration-dependent effect on cell proliferation: the cell viability in the 0.2 GO group showed no significant difference compared to the 0 GO group, consistently maintaining a high level; although the cell viability in the 1 GO group increased with the culture time, it was significantly lower than that in the 0 GO and 0.2 GO groups on the 5th day. This is speculated to be related to the physical stimulation caused by the aggregation of high-concentration GO and the mild oxidative stress induced by reactive oxygen species (ROS) generated from the residual oxygen-containing functional groups on the GO surface, exhibiting low toxicity [40,41].

On the 3rd and 5th days, the relative proliferation rates of cells in the 1 GO group reached 95.22% and 102.76%, respectively, both meeting the toxicity grading standard (>75%). In summary, when the GO content does not exceed 0.5 mg/mL, the hydrogel exhibits excellent cell compatibility, providing key experimental evidence for its safe application as a biological dressing.

Figure 4c visually characterizes the adhesion and spreading behavior of cells on hydrogels with different GO contents through fluorescence staining of the cytoskeleton (F-actin, red) and cell nucleus (DAPI, blue). In the 0 GO, 0.2 GO, and 0.5 GO groups, the number of cells is sufficient, with typical spindle or polygonal shapes. The cytoskeleton is clear, and pseudopodia are fully extended. The red fluorescence is evenly distributed and of high intensity, indicating good cell vitality and mature cytoskeleton development. However, the 1 GO group had relatively fewer cells, with a more rounded shape and limited pseudopod extension, indicating low cytotoxicity of GO, consistent with the relative proliferation rate results. Figure 4d SEM morphology further confirms that cells can tightly adhere to the surface of the hydrogel and extend filamentous pseudopodia to achieve effective anchoring and crawling. The combined results of fluorescence staining and SEM indicate that the prepared DA/CS/GO/PVA-coated hydrogel exhibits excellent cell compatibility. The appropriate addition of GO can provide a suitable microenvironment for cell growth, significantly promoting cell adhesion and spreading.

#### 3.3.3. Antioxidant Experiment

**Figure 5:**
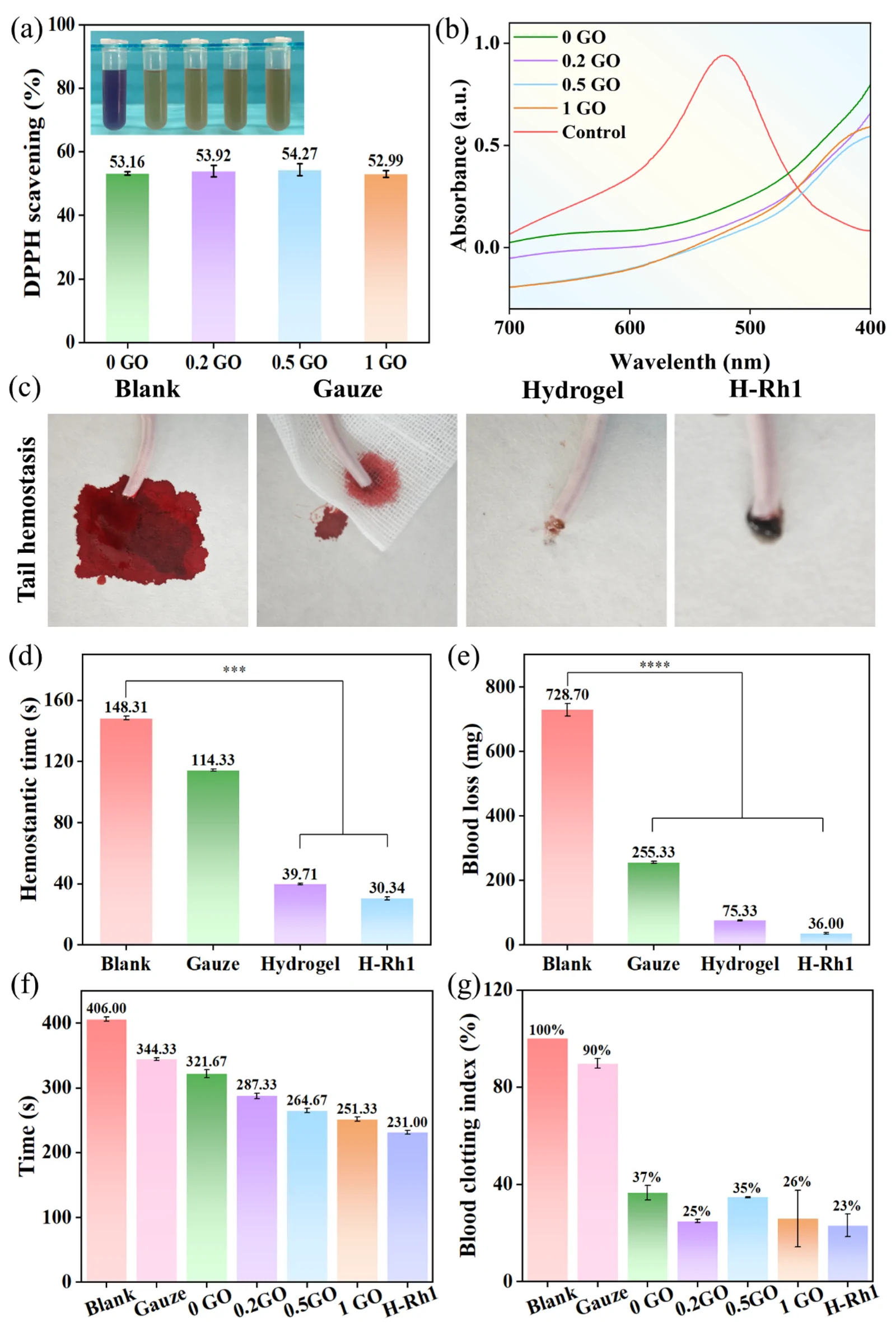
(**a**) Analysis of DPPH radical scavenging rate and antioxidant effect of hydrogels; (**b**) UV absorption spectrum of DPPH solution; (**c**) hemostasis effect of mouse tail amputation; (**d**) hemostasis time; (**e**) total bleeding amount; (**f**) coagulation time; (**g**) coagulation index BCI. (*** *p* < 0.001, **** *p* < 0.0001).

Figure 5a, quantitative analysis, shows that DA/CS/GO/PVA composite hydrogels all exhibit significant DPPH radical scavenging ability, with scavenging rates all above 50%, among which the 0.5 GO group shows the best effect (54.27%). This indicates that the matrix itself possesses good hydrogen and electron donation capabilities, and an appropriate amount of GO can provide synergistic sites through π–π interactions and hydrogen bonding. However, excessive GO tends to aggregate, weakening this activity, resulting in a slight decrease in the scavenging rate (52.99%). The results indicate that an appropriate amount of GO exhibits a synergistic antioxidant effect with the polymer network. As shown in Figure 5a, the deep purple DPPH solution decolorizes to light yellow after reacting with the hydrogel, further confirming that the hydrogel material possesses reliable and controllable antioxidant properties [42]. Figure 5b shows that the UV absorption spectrum provides direct spectral evidence for the quantitative analysis of antioxidant activity. The control group DPPH solution exhibits a strong characteristic absorption peak at 517 nm, while the absorbance at this wavelength is significantly reduced in all hydrogel groups, indicating effective radical scavenging. Among them, the 0.5 GO group shows the most pronounced decrease in peak intensity, consistent with the highest scavenging rate results, confirming the superior radical scavenging efficiency of this component.

#### 3.3.4. Mouse Tail Amputation Hemostasis Experiment

Based on the analysis of the above results, the 0.5 GO group hydrogel, which showed relatively the best performance, was selected for the next in vivo experiment. To further verify the hemostatic effect of DA/CS/GO/PVA-Rh1 (H-Rh1) hydrogel in a real physiological environment, a mouse tail amputation hemostasis experiment was conducted. Figure 5c shows the hemostatic effects of different experimental groups on the tail amputation wound in the tail amputation model. The blank control group showed continuous bleeding from the wound, and the gauze group showed improvement but still had significant oozing, while both the hydrogel without drugs (Hydrogel) and the H-Rh1 group formed stable blood clots on the wound surface, indicating their excellent hemostatic capabilities. Figure 5d shows the analysis of hemostasis time, with the hemostasis times for the H group and the H-Rh1 group being 39.71 s and 30.34 s, respectively. This demonstrates that the hydrogel loaded with Rh1 can rapidly adhere to the wound surface, concentrate the blood, and stimulate coagulation, significantly enhancing hemostatic efficiency.

Figure 5e shows the total amount of tail bleeding, where the H-Rh1 group had the lowest bleeding amount of only 36 mg, lower than all other experimental groups. This is mainly attributed to the wet surface adhesion provided by DA in the DA/CS/GO/PVA hydrogel and the positive charge of CS promoting red blood cell aggregation [43]. However, the detailed cellular and molecular mechanisms involved in the hemostatic process require further investigation.

#### 3.3.5. Coagulation Experiment

Figure 5f quantitatively evaluates the pro-coagulation ability of DA/CS/GO/PVA composite hydrogel dressings with different GO contents and the H-Rh1 group through in vitro whole blood coagulation time tests. The coagulation time of the Blank group was 406 s, indicating that the natural coagulation process of blood is slow without external intervention. The coagulation time of the Gauze group was 344.33 s, which, although lower than that of the Blank group, remained at a high level, consistent with its function of primarily providing a physical barrier rather than actively activating coagulation. In contrast, all drug-free hydrogel groups and the H-Rh1 group exhibited significantly better pro-coagulation performance than the Blank and Gauze groups, and the coagulation ability showed a clear positive correlation with the GO content. Figure 5g shows the BCI coagulation index for each experimental group. BCI is a key indicator for evaluating coagulation; the lower the BCI value, the better the coagulation effect. The BCI values of all hydrogel groups were significantly lower than those of the Blank and Gauze groups, indicating that the GO lamellar structure rapidly absorbs plasma moisture through strong hydrophilicity and capillary action, efficiently concentrating platelets, red blood cells, and coagulation factors locally. Combined with the positive charge of CS and the adhesion of DA, this synergistically accelerates thrombin generation and fibrin network formation, significantly enhancing hemostatic efficiency.

#### 3.3.6. Photothermal Antibacterial Experiment

(1) “Photothermal Switch” Effect

**Figure 6:**
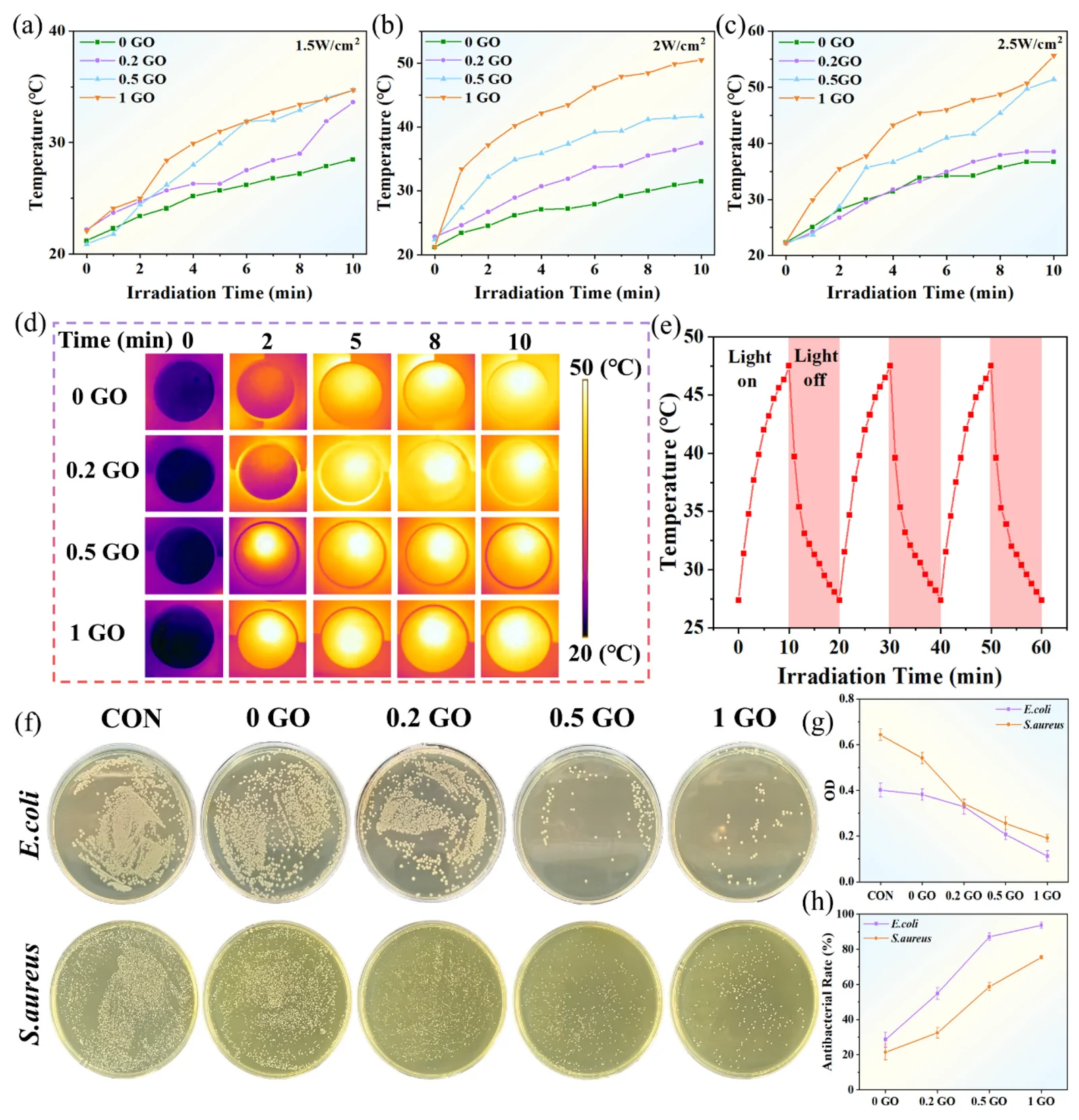
Antibacterial Performance of Stimulus-Responsive Hydrogels: (**a**–**c**) time–temperature curves under different power densities; (**d**) NIR irradiation heating effect of the hydrogel; (**e**) NIR “switch effect” of the 0.5 GO group hydrogel; (**f**) in vitro antibacterial images of *E. coli* and *S. aureus* on TSA plates with the hydrogel; (**g**) OD value curves of *E. coli* and *S. aureus* in different samples; (**h**) antibacterial rates of different samples against *E. coli* and *S. aureus*.

Figure 6a–c show the time–temperature curves of DA/CS/GO/PVA composite hydrogels under different near-infrared (NIR) power density (1.5, 2.0, 2.5 W/cm^2^) stimulations. Each group exhibited a rapid temperature rise at the beginning of irradiation, followed by a gradual stabilization; under the same power density, the heating rate and equilibrium temperature of the hydrogel significantly increased with the GO content due to the excellent photothermal conversion effect of GO in the near-infrared region. Under the condition of 2.0 W/cm^2^ for 10 min, the equilibrium temperature of the 1 GO group can reach approximately 55 °C, while the 0.5 GO group reaches 51 °C; 1.5 W/cm^2^ and 2.5 W/cm^2^ are difficult to meet the treatment requirements due to being too low and too high, respectively. As the power density increases, the time for the hydrogel to reach the same temperature decreases, and the equilibrium temperature rises, indicating that its photothermal performance can be synergistically regulated by the GO content and irradiation parameters, proving that GO is the core component of photothermal performance. By adjusting the irradiation intensity, the temperature rise of the hydrogel can be effectively controlled, thereby achieving precise thermal therapy for the wound area [44].

Figure 6d visually presents the surface temperature distribution changes in hydrogels with different GO contents under NIR irradiation through infrared thermal imaging. After 2 min of irradiation, the high GO content hydrogels (0.5 GO, 1 GO) had higher surface temperatures and a more uniform distribution, indicating that GO was well dispersed in the matrix. However, the 0 GO group and the 0.2 GO group appeared darker overall, indicating lower temperatures. The thermal imaging results are highly consistent with the heating curves, intuitively confirming that the GO content is positively correlated with the photothermal response performance of the hydrogel, providing direct evidence for its application in localized hyperthermia.

In order to evaluate the photothermal stability of the hydrogel, cyclic heating and cooling experiments were conducted. When the 0.5 GO group was irradiated with NIR, the temperature of the hydrogel rapidly rose to over 40 °C, and then naturally cooled down to about 27 °C (Figure 6e). This process was repeated over three cycles, and the results showed that the DA/CS/GO/PVA composite hydrogel maintained stable photothermal performance over multiple cycles, demonstrating its reliability and reusability in practical applications.

(2) Antibacterial Performance

Figure 6f visually demonstrates the in vitro antibacterial effects of DA/CS/GO/PVA composite hydrogels against Escherichia coli (*E. coli*) and Staphylococcus aureus (*S. aureus*) using the plate colony counting method. It shows that as GO gradually increases, the number of colonies on the two plates significantly decreases. Among them, the 1 GO group exhibited the strongest antibacterial effect, confirming the antibacterial properties of the smearable hydrogel. As shown in Figure 6g, the OD value results of the samples are highly consistent with the observations of the colony plates. Among them, the OD value of the CON group is the highest, indicating the maximum bacterial concentration and vigorous growth. As the GO content increases, the OD values representing bacterial concentration continue to decrease, demonstrating that the hydrogel effectively inhibits bacterial proliferation. Moreover, under the same GO content, the OD value for *S. aureus* is generally slightly lower than that for *E. coli*, suggesting that this hydrogel may have a superior inhibitory effect on Gram-positive bacteria. The OD value curve shows a smooth decline, indicating that the antibacterial effect of GO exhibits good dose-dependence.

The antibacterial rate in Figure 6h quantifies the antibacterial performance of the hydrogel. The antibacterial rates of all GO-containing hydrogels are significantly higher than those of the 0 GO group, and the antibacterial rates increase with the GO content. Specifically, the 0.5 GO group shows antibacterial rates of 58.71% and 87.00% against the two types of bacteria, respectively, while the 1 GO group increases to 75.37% and 93.62%, demonstrating strong bactericidal capabilities. The antibacterial rate curve clearly indicates that GO is a key functional component of the material’s antibacterial activity. Its synergistic effect with the inherent antibacterial properties of CS [45], through a combination of physical disruption (GO nanosheet edges cutting cell membranes) and chemical damage (inducing reactive oxygen species production) [46], significantly enhances the material’s antibacterial performance, meeting the demand for high-efficiency antibacterial wound dressings.

### 3.4. Wound Analysis

#### 3.4.1. Wound Healing Efficiency Analysis

**Figure 7:**
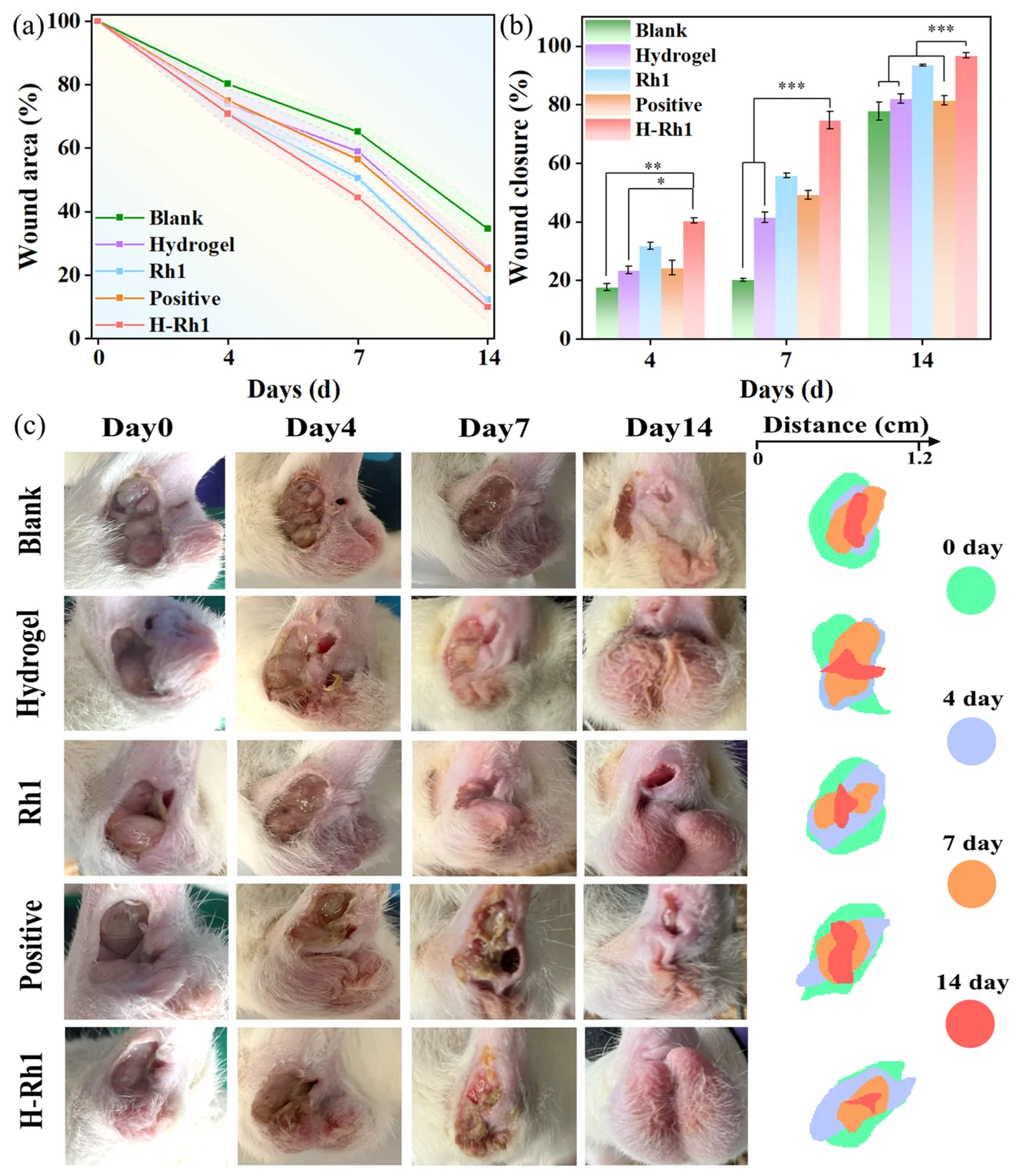
Healing effect of perianal ulcer wounds: (**a**) relative wound area at days 0, 4, 7, and 14; (**b**) wound healing rate; (**c**) representative images of wound healing and traces of wound closure during treatment. (* *p* < 0.05, ** *p* < 0.01, *** *p* < 0.001).

Figure 7a shows the trend of relative wound area changes over 14 days for different treatment groups. The wound area in all groups decreased over time, with the H-Rh1 group exhibiting the fastest healing rate. The wound area in the H-Rh1 group showed a significant continuous decline from the initial stage, reaching a relative wound area rate of only 9.89% by day 14, nearing complete healing. The Rh1, Positive, and Hydrogel groups also demonstrated good healing effects, with relative wound area rates of 12.22%, 21.84%, and 22.42% on day 14, respectively. The Blank group, due to the lack of any intervention, had the slowest healing rate, particularly after day 7, when the trend of wound area reduction was the slowest. Therefore, the biomimetic stimulus-responsive hydrogel dressing (H-Rh1) can significantly accelerate the wound healing process.

Through quantitative analysis of wound healing rates, the healing effects of each group were further verified. As shown in Figure 7b, the healing rate of the Blank group was only 77.77% by the 14th day. The Positive group demonstrated a better healing effect than the Blank group on the 4th and 7th days, but its healing rate on the 14th day was 81.49%, indicating that it has some repair ability, but the effect is not significant. The wound healing rate of the Hydrogel group was slightly better than that of the Blank and Positive groups, with a healing rate of approximately 82.06% by the 14th day. The healing rate of the Rh1 group on the 14th day was 93.44%, second only to the H-Rh1 group. The most remarkable healing effect was observed in the H-Rh1 group, whose healing rate was significantly higher than that of the other experimental groups at all time points. On the 14th day, the healing rate was approximately 96.78%. This group exhibited an accelerated trend in the early healing phase and maintained the fastest healing rate during the proliferation phase. The introduction of GO not only enhances the multifunctionality of the hydrogel in terms of hemostasis, coagulation, antioxidation, and antibacterial properties, but also provides a stable and moisturizing physical microenvironment for the wound. Under NIR irradiation, the hydrogel dressing effectively controls infection and promotes the smooth transition of the inflammatory phase. At the same time, the larger specific surface area of GO, acting as a carrier, effectively slows down the burst release of Rh1, which possesses anti-inflammatory and antioxidant properties, and promotes fibroblast proliferation. Their synergistic effect makes the prepared composite hydrogel dressing exhibit excellent application potential in promoting ulcer wound healing.

Through in vivo animal experiments, the wound healing process of different experimental groups on the ulcer model was directly recorded. As shown in Figure 7c, the wound area in all groups decreased over time (days 0, 4, 7, and 14). The healing process of the Blank group is the slowest, with obvious ulcerated wounds still visible by day 14 and incomplete wound edge contraction, indicating limited natural healing ability; the Positive group shows some promoting effect on healing by day 7, but the wound is still not fully closed by day 14; the healing effects of the Hydrogel group and the Rh1 group are similar, both better than the Blank group but lower than the H-Rh1 group. The most significant healing effect is observed in the H-Rh1 group: by the 7th day, a large amount of fresh granulation tissue can be seen in the lesion area of this group, and by the 14th day, the wound has almost completely epithelialized, leaving only minimal traces. The quantification chart of the wound closure on the right side also visually demonstrates that the healing effect of the H-Rh1 group is the best. This exceptional healing-promoting ability is attributed to the multiple synergistic effects of DA/CS.

#### 3.4.2. Histological Analysis of Wounds

**Figure 8:**
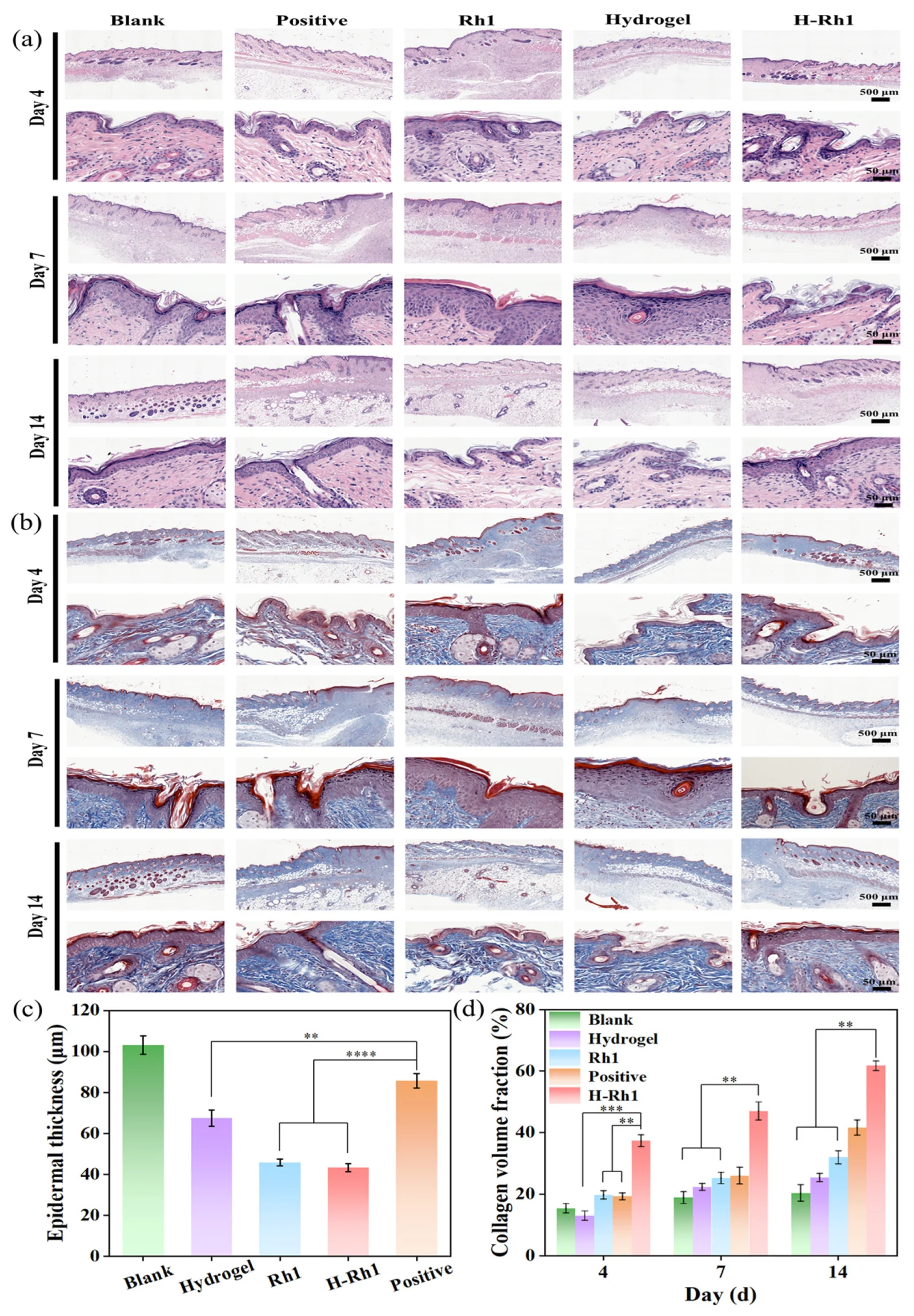
Histological characteristics of the wound: (**a**,**b**) H&E and Masson staining of rat wound skin tissue; (**c**) statistical analysis of wound skin thickness; (**d**) quantitative analysis of collagen deposition. (** *p* < 0.01, *** *p* < 0.001, **** *p* < 0.0001).

Figure 8a shows the histological changes in wounds in each group on days 4, 7, and 14. On day 4, the H-Rh1 group exhibited reduced inflammatory cell infiltration and enhanced early granulation tissue formation compared with the control groups. By day 7, the H-Rh1 group showed more mature granulation tissue with abundant neovascularization and fibroblast proliferation. On day 14, the H-Rh1 group achieved improved re-epithelialisation and collagen organisation, whereas the control groups exhibited incomplete tissue repair. These results demonstrate that the DA/CS/GO/PVA-Rh1 hydrogel effectively promotes tissue regeneration and accelerates wound healing.

Quantitative analysis in Figure 8c,d further confirmed the histological findings. On day 14, the H-Rh1 group exhibited a more appropriate epidermal thickness (43.36 μm) compared with the Blank (103.15 μm) and Positive groups (85.75 μm), indicating improved epithelial regeneration. Moreover, the H-Rh1 group showed the highest collagen volume fraction, reaching 61.73% on day 14, suggesting enhanced collagen deposition and tissue remodeling. These results demonstrate that the DA/CS/GO/PVA-Rh1 hydrogel not only accelerates wound closure but also improves tissue regeneration quality.

## 4. Conclusions

In this study, a mussel-inspired multifunctional hydrogel dressing (DA/CS/GO/PVA-Rh1) was successfully developed for perianal ulcer treatment by integrating wet adhesion, controlled drug release, antioxidant activity, photothermal antibacterial capability, and tissue regeneration promotion. The optimized hydrogel containing 0.5 mg/mL GO exhibited favorable mechanical stability, sustained Rh1 release behavior, excellent cytocompatibility, and low hemolysis (<2.45%). Moreover, the hydrogel demonstrated strong wet-tissue adhesion, with a maximum shear strength of 29.49 kPa, and exhibited effective antibacterial activity against *E. coli* and *S. aureus* under 808 nm near-infrared irradiation. In the rat perianal ulcer model, the H-Rh1 hydrogel significantly accelerated wound repair, achieving a wound closure rate of 96.78% on day 14. Histological analyses further confirmed enhanced re-epithelialisation, collagen deposition, and tissue remodeling after treatment. Although these results demonstrate the potential of this biomimetic hydrogel for complex wound management, further investigations involving larger animal models and clinical translation are required. Overall, this work provides a promising strategy for developing multifunctional hydrogel dressings for difficult-to-heal perianal ulcers.

## Data Availability

The data that support the findings of this study are available from the corresponding author upon reasonable request.
